# Intense L-Band Solar Radio Bursts Detection Based on GNSS Carrier-To-Noise Ratio Decrease over Multi-Satellite and Multi-Station

**DOI:** 10.3390/s21041405

**Published:** 2021-02-17

**Authors:** Fan Yang, Xuefen Zhu, Xiyuan Chen, Mengying Lin

**Affiliations:** School of Instrument Science and Engineering, Southeast University, Nanjing 210096, China; 220183292@seu.edu.cn (F.Y.); chxiyuan@seu.edu.cn (X.C.); 230198865@seu.edu.cn (M.L.)

**Keywords:** global navigation satellite system, solar radio bursts, carrier-to-noise ratio, detection rate

## Abstract

Intense solar radio bursts (SRBs) can increase the energy noise and positioning error of the bandwidth of global navigation satellite system (GNSS). The study of the interference from intense L-band SRBs is of great importance to the steady operation of GNSS receivers. Based on the fact that intense L-band SRBs lead to a decrease in the carrier-to-noise ratio ($C/N_{0}$) of multiple GNSS satellites over a large area of the sunlit hemisphere, an intense L-band SRB detection method without the aid of a radio telescope is proposed. Firstly, the valley period of a single satellite at a single monitoring station is detected. Then, the detection of SRBs is achieved by calculating the intersection of multiple satellites and multiple stations. The experimental results indicate that the detection rates of GPS L2 and GLONASS G2 are better than those of GPS L1 L5, GLONASS G1, and Galileo E1 E5. The detection rate of SRBs can reach 80% with a flux density above 800 solar flux unit (SFU) at the L2 frequency of GPS. Overall, the detection rate is not affected by the satellite distribution relative to the Sun. The proposed detection method is low-cost and has a high detection rate and low false alarm rate. This method is a noteworthy reference for coping with interference in GNSS from intense L-band SRBs.

## 1. Introduction

Global navigation satellite system (GNSS) is currently widely used in both military and civilian fields [1,2]. It is extremely important to keep this system free of interference. Types of interference with GNSS include multipath [3,4], ionospheric scintillation [5], spoofing [6,7], electromagnetic interference, satellite signal anomalies, and intense L-band solar radio bursts (SRBs) [8]. SRB is a strong signal in the microwave band from solar plasma radiation and cyclotron synchrotron radiation. The entire radiation bandwidth extends from the millimeter to the kilometer wave band. L-band SRB (1415 MHz) interferes with the GNSS signal most significantly due to the fact that it is close to GNSS frequencies, such as GPS (L1: 1575.42 MHz, L2: 1227.60 MHz, L5: 1176.45 MHz), GLONASS (G1: 1602 MHz, G2: 1246 MHz) and Galileo (E1: 1575.42 MHz, E5: 1191.795 MHz). Strong radio noise can cause the carrier-to-noise ratio ($C/N_{0}$) of a GNSS receiver to decrease, which directly affects the tracking and acquisition of the GNSS signal. In worse interference cases, cycle slip, loss of lock, interruption and other phenomena may occur [9].

Researchers have studied the impact of SRB on GNSS since 2005. Chen et al. [10] analyzed the phenomenon of GPS loss of lock due to a SRB event on 28 October 2003. They pointed out that the flux density threshold of the solar radio bursts affecting GPS signals is between 4000 and 12,000 solar flux unit (SFU). Cerruti et al. [11] analyzed the effects of several SRB events in December 2006 on the $C/N_{0}$ of GPS and found a strong correlation between $C/N_{0}$ and solar radio emission power. Through theoretical analysis, Demyanov et al. [12] found that SRBs with emission power of 1000 SFU or higher may lead to GPS or GLONASS tracking faults. The effect is especially prevalent at the L2 frequency, which is lower than the threshold proposed by Chen et al. [10]. Yue et al. [13] analyzed the influence of SRBs on GPS $C/N_{0}$ and concluded that the threshold value of solar radio burst effects on GNSS is approximately 1807 SFU. Sreeja et al. [14,15] studied the impact of the SRB event on 24 September 2011 on a single-point precise positioning service and the performance of GNSS receivers. Subsequently, Muhammad et al. [16] studied the event with higher sampling GPS data and evaluated the impact of SRBs on GPS signal characteristics, including the signal-to-noise ratio, loss of lock, and phase tracking. The study of [17] shows that SRBs are not limited to the vicinity of sunspot maxima. Huang et al. [18] specifically analyzed the influence of a SRB event on 13 December 2006 on GNSS performance and positioning error in several regions in China. The effects of the SRB event on 6 September 2017 on GNSS signals and the corresponding response of the ionosphere were studied in [19,20,21,22].

Recently, a number of automatic detection and classification methods for SRBs have been proposed. Ma et al. [23] proposed a multimodal deep learning method for SRB classification. An autoencoder (AE) together with structured regularization was used to enforce and learn the modality-specific sparsity and density of each modality. The proposed network can effectively learn the representation of the solar radio spectrum. Subsequently, Chen et al. [24] utilized a convolutional neural network (CNN) for the classification of solar radio spectra and obtained better experimental results than Ma et al. [23]. Zhang et al. [25,26] designed an event recognition-analysis system that can automatically detect solar type III radio bursts and mine burst information from the dynamic spectra observed by the Nancay Decameter Array (NDA). Singh et al. [8] proposed an automated method to detect SRBs. Although the method does not classify the types of radio bursts, it is able to discriminate between dynamic spectra with and without SRBs.

At present, although there have been some SRB automatic recognition algorithms, most of the methods utilize radio telescopes to obtain observation data and then classify SRBs according to the spectra. As radio telescopes are expensive and sparsely distributed, we urgently need a method to efficiently detect SRBs in real time without utilizing radio telescopes. Since 2005, many studies have focused on the impact of SRBs on GNSS receiver observation data. However, there is a lack of specific algorithms or experimental verification to detect SRBs using its impact on GNSS receiver observation data. Despite the impact of SRBs on GNSS cannot be completely eliminated, some measures can be taken to reduce or alleviate the interference caused by SRBs. Furthermore, real-time and accurate identification of SRBs is an essential prerequisite for suppressing the interference.

In this paper, intense L-band SRB events are detected using the fact that SRBs lead to a decrease in the $C/N_{0}$ of several GNSS satellites over a large area of the Earth’s surface. Other interference, such as ionospheric scintillation and multipath, may also cause a decrease in the $C/N_{0}$, but this phenomenon does not simultaneously occur on multiple satellites over a large area of the sunlit hemisphere, which is a significant feature that distinguishes SRBs from other types of interference. During the detection process, the ‘‘falling moments’’ and ‘‘rising moments’’ are first selected to determine the valley period of a single satellite at a single monitoring station. Then, the detection of a single station is obtained by comparing multiple satellites over the station. Finally, the detection of intense L-band SRBs is calculated by comparing multiple stations. The proposed method, which does not rely on radio telescopes, has a high recognition rate and low false alarm rate, is low-cost, and offers all-weather real-time monitoring.

## 2. Effect of SRBS on GPS Receiver Noise Floor

Solar flares produce an increase in solar noise, which is measured in units of SFU, 10^−22^ W m^−2^ Hz^−1^, or −220 dBWHz^−1^. These SRBs are wideband noise, which increase the noise power of the receiver and result in a lower $C/N_{0}$. Measuring the solar radio emission power at the GPS receiver antenna output is critical to the steady operation of the GPS system.

The antenna directive gain is defined as the ratio between the power of the actual antenna P(β) and the power of the signal generated by the ideal radiation unit $P_{0}$ at the same point in space under the conditions of equal input power. This parameter quantitatively describes the degree to which an antenna radiates the input power, which can be defined as

(1) $$D(\beta )=P(\beta )/P_{0},$$

where β is the elevation angle of the GPS signal.

Obviously, the directive gain is closely related to the elevation angle of the antenna. The gain is usually computed in units of dB and expressed as G(β)=10⋅lg(D(β)). The results [27] for the gain corresponding to different elevation angles are shown in Table 1.

A GPS antenna needs to be able to receive as many GPS signals as possible so that its gain is very low in all directions. Moreover, the reception cross-section of the GPS antenna is low, especially near the horizon. The antenna effective area is defined as

(2) $$A_{e}(\beta )=\frac{4\pi \cdot D(\beta )}{\lambda^{2}},$$

where λ is the wavelength of the received signal.

The calculated results of the antenna effective area $A_{e}(\beta )$ for different elevation angles at GPS frequency bands are shown in Table 2.

The radio emissions of a solar flare can be taken as Gaussian white noise for GPS signals. The solar radio emission intensity is constant within the bandwidth $\Delta F_{n}$, so the radio emission power within the frequency bands of GPS signal can be calculated in a similar way and is defined as

(3) $$P_{n}=\Delta F_{n}\cdot N_{0}.$$

Therefore, without considering the polarization loss and atmospheric attenuation, the solar radio noise at the receiver antenna output can be expressed as [12]

(4) $$P_{n}=\Delta F_{n}\cdot k\cdot N_{0}\cdot A_{e}(\beta ),$$

where k is the rate of the solar radio emission flux in terms of SFU and $N_{0}=-10^{-22}{\;W\;m}^{-2}{\;\mathrm{Hz}}^{-1}$.

Finally, the solar radio noise power at the receiving antenna output for solar elevation angles $>15^{{}^{\circ}}$ at the central solar radio emission frequency f=1415 MHz (we assumed λ=0.212 m, $A_{e}=2.253\times 10^{-3}m^{2}$) is calculated and shown in Table 3 [12]. Note that the solar radio noise is computed in units of dBW, and the front-end passband of the GPS receiver radio path ($\Delta F_{\mathrm{GPS}}$) is 3 MHz [27].

## 3. Methodology

**Figure 1:**
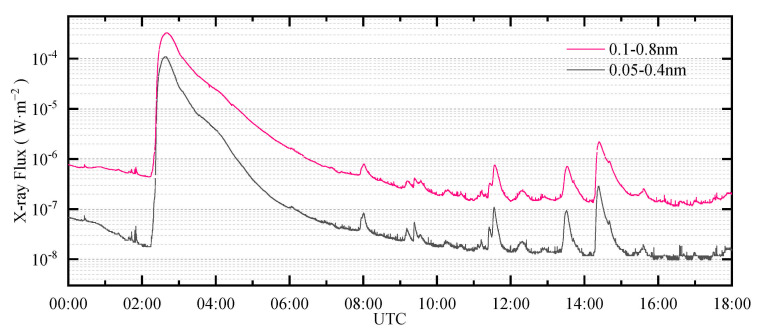
Solar X-ray flux measured by the Geostationary Operational Environmental Satellite (GOES) Solar X-ray Imager during the solar flare that occurred on 13 December 2006. The flare occurred from 02:14 to 02:57 (UTC), and the peak time was 02:40 (UTC). The sampling period was 3 s. The solar X-ray flux data were provided by the National Geophysical Data Center (https://satdat.ngdc.noaa.gov/sem/goes/data/, accessed on 6 January 2021).

Intense L-band SRBs can affect the GNSS observation data in a large area near the subsolar point, including through a decrease in $C/N_{0}$, an increase in Geometric Dilution Precision (GDOP) and positioning error. In the worse cases, it can also lead to the loss of lock. The $C/N_{0}$ shows the most obvious reaction to intense L-band SRBs, especially when an event is not severe. Figure 1 shows the solar X-ray flux measured by the Geostationary Operational Environmental Satellite (GOES) Solar X-ray Imager during an X3.4-level X-ray solar flare on 13 December 2006. According to the data, the flare occurred from 02:14 to 02:57 (UTC), and the peak time was 02:40 (UTC). This solar flare was accompanied by an intense L-band SRB that had a profound impact on the performance of GPS.

**Figure 2:**
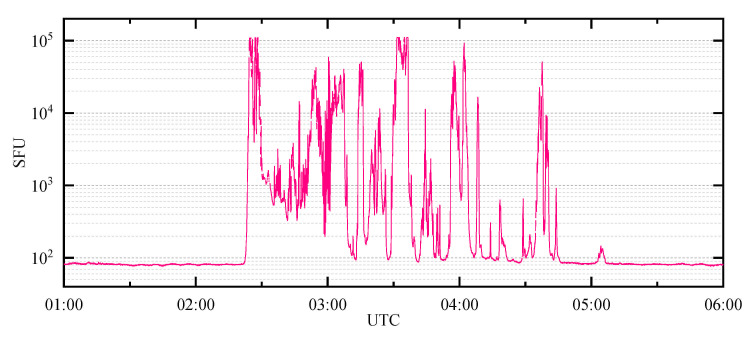
Solar radio flux at 1415 MHz observed at station Sagamore Hill provided by radio solar telescope network (RSTN) during the intense L-band solar radio bursts (SRB) event that occurred on 13 December 2006. The sampling frequency was 1 Hz, and the burst period was mainly approximately 02:30, 03:30 and 04:00 (UTC).

Figure 2 shows the L-band (1415 MHz) solar radio emission power observed at station Sagamore Hill in America during this SRB event. The data were obtained from the radio solar telescope network (RSTN), and the sampling frequency was 1 Hz. This SRB event occurred between 02:20:00 and 04:45:00 (UTC), and the peak flux value reached 1.1 × 10^5^ SFU, which was more than 1000 times of the normal value (<100 SFU).

**Figure 3:**
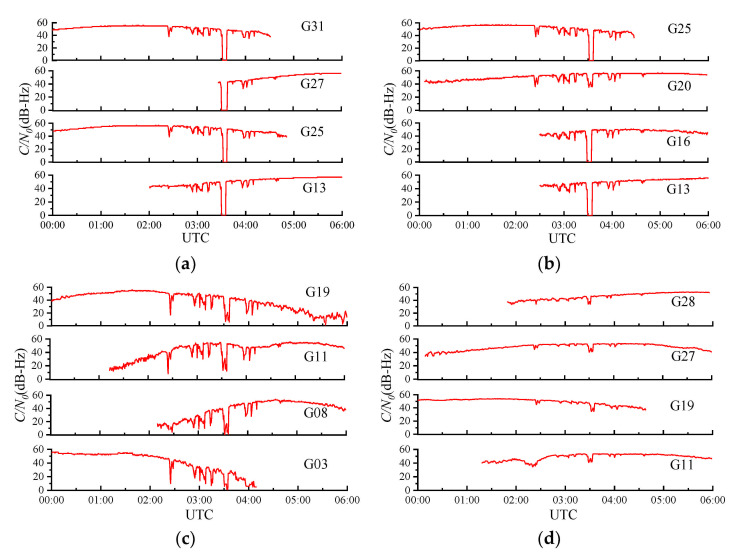
The $C/N_{0}$ values at the GPS L1 frequency for International Global Navigation Satellite System Service (IGS) stations CEDU (**a**), ALIC (**b**), TWTF (**c**) and KUNM (**d**) during the intense L-band SRB event that occurred on 13 December 2006. The solar elevation angles of the four stations were 80.7°, 84.9°, 42.5° and 36.0° respectively. The sampling period was 30 s.

We select stations CEDU and ALIC in Australia as well as stations TWTF and KUNM in China to analyze the influence of the SRB on the $C/N_{0}$ on 13 December 2006. The solar elevation angles of the four stations were 80.7°, 84.9°, 42.5° and 36.0°, respectively. Figure 3 shows the decrease in the $C/N_{0}$ of different satellites at each station for the GPS L1 frequency. The data were provided by the International GNSS Service (IGS), and the sampling period was 30 s. Figure 3 shows that the $C/N_{0}$ of each station decreased to varying degrees during the SRB.

The influence of the SRB on the $C/N_{0}$ of the GNSS receiver increased with increasing solar elevation angle, which is consistent with the conclusions in the literature [18]. Station ALIC and station CEDU in Australia were closer to the subsolar point, and consequently, their $C/N_{0}$ values showed a stronger decline. The $C/N_{0}$ values of the four satellites at station CEDU decreased sharply, and all of them had a loss of lock at 03:31 (UTC), with a maximum duration of approximately 450 s. In contrast, none of the four satellites at station KUNM had a loss of lock, despite the significant decline in $C/N_{0}$ of approximately 12 dB-Hz. However, the fading trends of all the GPS satellites at the different stations were almost the same. In other words, most satellites had valleys at the same time despite their different degrees of decline, especially at approximately 3:31 (UTC).

Based on the above analysis, we propose an intense L-band SRB detection method using the fact that SRBs lead to a decrease in the $C/N_{0}$ values for signals from several GNSS satellites over a large area of the Earth’s surface. In this process, data preprocessing is initially performed, and the specific details are as follows:

- The solar elevation angle of all the IGS stations is calculated to select the stations close to the subsolar point.
- The data of improperly working receivers are excluded, such as some stations with incomplete data.
- The data type of $C/N_{0}$ is set to an integer to eliminate the impact of the differences in the data precision of various types of receivers on the results.
- The elevation mask angle is set to 10°. Note that the elevation mask angle is lower than normal to increase the amount of observation data. Due to the fact that GPS L5 and GALILEO E5 include few satellites that can be observed at the same time from the data provided by the IGS. In addition, multipath cannot lead to a simultaneous decrease in the $C/N_{0}$ values of multiple satellites over a large area close to the subsolar point. Hence, the proposed method can prevent the impact of multipath to some extent, which is reflected in the low false alarm rate in the subsequent experiments.

Then, the valley periods of a single satellite at a single station are calculated, and finally, the detection result of an intense L-band SRB is obtained by comparing multiple satellites and multiple stations.

### 3.1. Detection of a Single Satellite

The detection procedure for a single satellite includes two significant modules: finding the falling/rising moments and determining the valley periods.

#### 3.1.1. Determination of the ‘‘Falling Moments’’ and ‘‘Rising Moments’’

**Figure 4:**
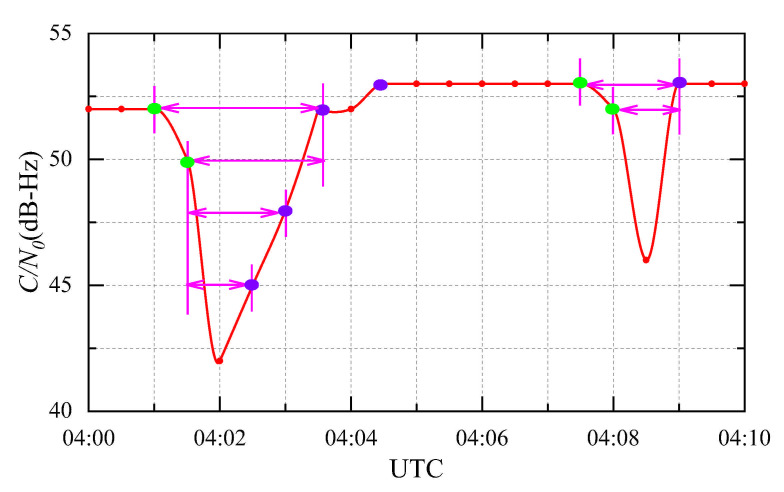
The $C/N_{0}$ of GPS L1 G13 at IGS station CEDU during an intense L-band SRB event that occurred on 13 December 2006. The sampling period was 30 s.

We define the moment at which $C/N_{0}$ is higher than that at the next moment as the falling moment. This is denoted as $a_{i}(1\leq i\leq N)$, where N is the number of falling moments. The set of falling moments, which is denoted as A, can be expressed as $A=\left\{a_{i}|1\leq i\leq N\right\}$. Likewise, the moment at which $C/N_{0}$ is higher than that at the previous moment is defined as the rising moment and denoted $b_{j}(1\leq j\leq M)$. For this expression, M is the number of rising moments, and the set of rising moments, which is denoted as B, can be expressed as $B\;=\;\left\{b_{j}|1\leq j\leq M\right\}$. To illustrate the model with an example, the $C/N_{0}$ values of G13 at the GPS L2 frequency observed at station CEDU during the SRB event on 13 December 2006 are shown in Figure 4. The green points are the falling moments, while the purple points represent the rising moments.

#### 3.1.2. Determination of the Valley Period

The valley period is defined as the period between the falling moment and the rising moment and must be satisfied as follows:

- The time of the rising moment is higher than the falling moment.
- The $C/N_{0}$ at any time in this period is less than the smaller of that at the falling moment and that at the rising moment.

The set of valley periods, which is denoted as P, can be expressed as

(5) $$P=\left\{\left(a_{i},b_{j}\right)\left|\begin{array}{c} a_{i}<b_{j},\forall t\in \left(a_{i},b_{j}\right),\\ {C/N_{0}}_{t}<\mathrm{Min}\left({C/N_{0}}_{a_{i}},{C/N_{0}}_{b_{j}}\right) \end{array}\right.\right\},$$

where t is the moment within the period ($a_{i}$,$b_{j}$). ${C/N_{0}}_{t}$, ${C/N_{0}}_{a_{i}}$ and ${C/N_{0}}_{b_{j}}$ represent the ratio of the carrier to noise at moments t, $a_{i}$ and $b_{j}$, respectively. As an example, the valley periods in Figure 4 are marked in magenta. Additionally, we show two valley periods in Figure 4 to indicate that the proposed method can effectively distinguish various valley periods.

Similarly, the valley period of each satellite over the same station at a selected frequency can be calculated and defined as $P_{s}(1\leq s\leq l)$, where s and l represent the corresponding satellite and the number of observed satellites, respectively.

### 3.2. Intersection of Different Satellites at the Same Monitoring Station

The valley periods common to multiple satellites at a single station are denoted G and can be expressed as

(6) $$G=P_{1}\cap P_{2}\cap \cdots \cap P_{l},$$

where $P_{s}(1\leq s\leq l)$ represents the valley period set of satellite s.

Similarly, the valley periods common to all satellites at each station can be calculated and defined as $G_{k}(1\leq k\leq m)$, where m is the number of stations and $G_{k}$ is the valley period set at station k.

### 3.3. Intersection of Multiple Monitoring Stations

Likewise, the valley periods common to multiple stations are denoted by U and can be expressed as

(7) $$U=G_{1}\cap G_{2}\cap \cdots \cap G_{m}.$$

All the moments in the set of U are the final detection results.

## 4. Results and Discussion

In this work, we first study the influence of the number of satellites and the number of stations on the detection results. Based on these results, the influence of the satellite distribution on the detection rate is analyzed. Finally, the detection rates at different frequency bands of different GNSS systems are compared.

All the GNSS observation data used in the experiment are taken from the IGS data center. The sampling period is 30 s, and each sampling point is counted as an event. The solar radio emission power value at 1415 MHz provided by RSTN is compared with our detected samples. The emission power values of different magnitudes are separately counted. Accordingly, the detection rate, which is used to represent the detection performance, is defined as the ratio between the number of predicted SRB samples and the number of real samples (from RSTN) in each emission power interval. We define the minimum emission power corresponding to the detection rate of 80% as the detection threshold in the experiments.

Table 4 presents the detailed information of each station used in the experiments, including ID, latitude, longitude and country.

### 4.1. Detection of a Single Satellite

The data from station NNOR on 13 December 2006 are used for the detection of a single station. The detection period is 00:00:00–10:00:00 (UTC), and the time of the peak solar radio emission power is approximately 03:30:00 (UTC), with a solar incident angle of 77.1°. All satellites observed at the peak time are detected separately using the method described in Section 3.1. The valley period common to at least $N_{sat}$ satellites is judged as the detection result of a single station. In this section, different values of $N_{sat}$ are compared and analyzed.

**Figure 5:**
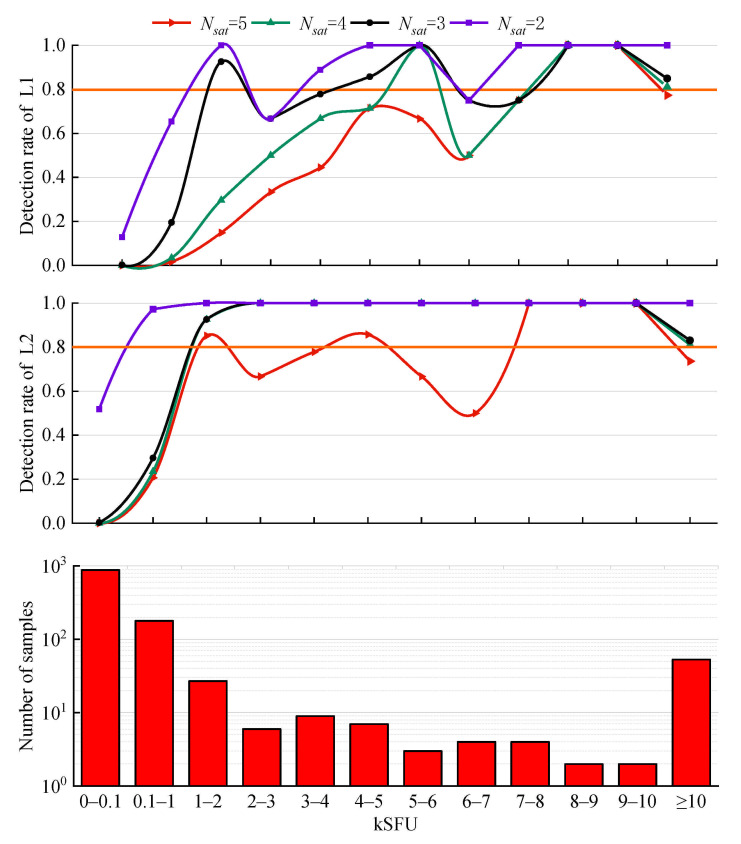
Detection rate of GPS L1 and L2 at IGS station NNOR during an intense L-band SRB event that occurred on 13 December 2006. The histogram shows the number of samples in each flux range.

Figure 5 presents the detection rates corresponding to different $N_{sat}$ for GPS. At both the L1 and L2 frequencies, the detection rate decreases with increasing $N_{sat}$ for any flux range due to the increasingly severe restriction. Moreover, regardless of the value of $N_{sat}$, the detection rate at the L2 frequency is significantly higher than that at the L1 frequency. A possible reason for this is that flux or power density at different frequencies of GNSS may have a big difference even for the same L-band SRB event [22].

In the case of $N_{sat}$ = 5, only a few flux ranges have a detection rate of ≥80%. For GPS L1, only 8–9 kSFU and 9–10 kSFU reach 80%; for GPS L2, only 1–2 kSFU, 4–5 kSFU, 7–8 kSFU, 8–9 kSFU, and 9–10 kSFU reach 80%. Overall, the detection rate is quite low in the case of $N_{sat}$ = 5.

Conversely, in the case of $N_{sat}$ = 2, the detection rate at both frequencies reaches the highest for all the flux ranges due to the less severe restriction. However, the detection rates of GPS L1 and L2 in the range of 0–100 SFU reach 12.87% and 51.82%, respectively. Note that the flux density under normal circumstances is below 100 SFU, and thus, the detection rate of this range represents the false alarm rate. As mentioned above, the false alarm rate is much higher in the case of $N_{sat}$ = 2.

Furthermore, in the case of $N_{sat}$ = 3 or $N_{sat}$ = 4, the detection rate in the range of 0–100 SFU is close to 0, with higher reliability. Regarding GPS L1, for the ranges of 5–6 kSFU, 7–8 kSFU, 8–9 kSFU, and 9–10 kSFU, the detection rate of $N_{sat}$ = 3 is equivalent to that of $N_{sat}$ = 4. However, the detection rate of $N_{sat}$ = 3 is significantly higher than that of $N_{sat}$ = 4 when the flux is lower. Regarding GPS L2, for the range of 0.1–1 kSFU, the detection rate of $N_{sat}$ = 3 is slightly higher than that of $N_{sat}$ = 4, with a difference of 6.15%, and the detection rates of the other ranges are equivalent. From the above discussion, we conclude that the optimal value of $N_{sat}$ is 3 due to its obvious advantage of low flux.

### 4.2. Analysis of Multiple Stations

In this section, to expand data coverage, we analyze additional three typical intense L-band SRB events. For each event, seven stations close to the subsolar point are selected. Table 5 lists the detailed information for each SRB event, including the period when the SRB occurred, the RSTN stations, the time of the peak of flux density, the IGS stations and the solar incident angle. Due to the incompleteness of the RSTN and IGS data, it is impossible to guarantee that the flux density data for different events are from the same RSTN station and that all seven stations are the stations closest to the subsolar point. For each SRB event, the valley period common to at least $N_{station}$ stations is judged as the final detection result. In addition, for each station, we take the value $N_{sat}$ = 3 from the optimization in Section 4.1.

**Figure 6:**
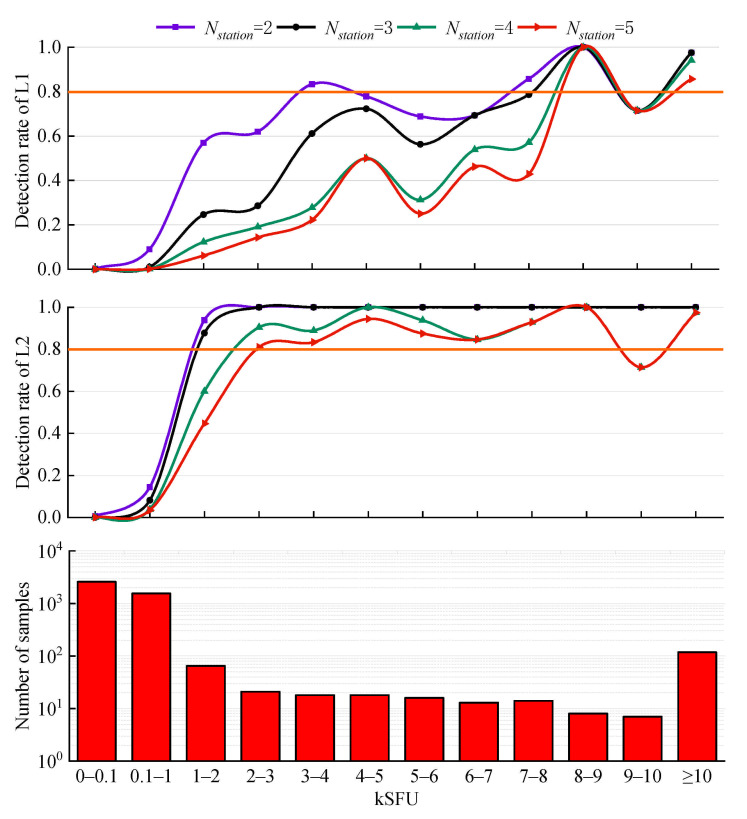
Detection rate for 4 intense L-band SRB events. The histogram shows the number of samples in each flux range.

Figure 6 presents the results corresponding to different values of $N_{station}$ for GPS. In addition to the time when the SRB occurred, the period detected in this experiment includes normal conditions. We can effectively test the false alarm rate based on the 2589 samples in the range of 0–100 SFU. Generally, the number of samples gradually decreases with increasing solar radio flow. The range of ≥10 kSFU is not further divided, as a flux density of more than 10 kSFU is quite easy to be detected.

With increasing $N_{station}$, the detection rates of both frequencies gradually decrease and reach approximately 0% in the range of 0.1–1 kSFU, proving that the proposed method has a low false alarm rate and high reliability. Furthermore, the low false alarm rate indicates that the proposed method can avoid the impact of multipath to some extent. In other words, if multipath interference is detected as SRB by mistake, the predicted samples are distributed in each flux range instead of most being in the ranges above 100 SFU.

The detection rate at GPS L2 is much higher than that at GPS L1, which is consistent with Figure 5. For GPS L1, there are a small number of flux ranges with a detection rate of 80%. In the case of $N_{station}$ = 2, only 3–4 kSFU, 7–8 kSFU, 8–9 kSFU, and 10 kSFU have detection rates ≥ 80%. However, for GPS L2, in cases of $N_{station}$ = 2, 3, and 4, the detection thresholds that meet the detection rate of 80% are in the ranges of 1–2 kSFU, 1–2 kSFU, and 2–3 kSFU, respectively. There is a sudden and obvious drop in the range of 9–10 kSFU at both the L1 frequency and L2 frequency. A possible reason for this is that merely 7 samples are in the range of 9–10 kSFU.

**Figure 7:**
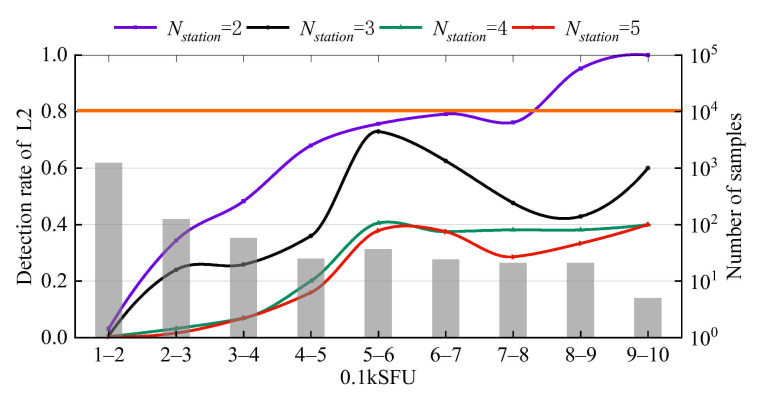
Detection rate in the range of 0.1–1 kSFU at the GPS L2 frequency for 4 intense L-band SRB events. The histogram shows the number of samples in each flux range.

Due to the detection rate at the L2 frequency being higher than that at the L1 frequency, we further divide the range of 0.1–1 kSFU at the L2 frequency for more in-depth analysis. As shown in Figure 7, there is a trend of fewer samples and a higher detection rate with increasing solar radio emission power. In the case of $N_{station}$ = 3, 4 or 5, there are no flux ranges with detection rates reaching 80%. However, in the case of $N_{station}$ = 2, the flux density threshold for a detection rate of 80% is in the range of 800–900 SFU. Specifically, the detection rates of 500–600 SFU, 600–700 SFU, and 700–800 SFU reach 80%, 75.68%, 79.17%, and 76.19%, respectively. As mentioned above, we can conclude that the optimal value of $N_{station}$ is 2.

### 4.3. Influence of Satellite Distribution on the Detection Rate

In this section, the GPS L1 L2 data of four typical SRB events in Table 5 are used to analyze the significance of the satellite distribution on the detection rate. The satellites are divided with respect to the incident direction of the Sun into two categories: ‘‘near-satellites’’ and ‘‘distant-satellites’’.

**Figure 8:**
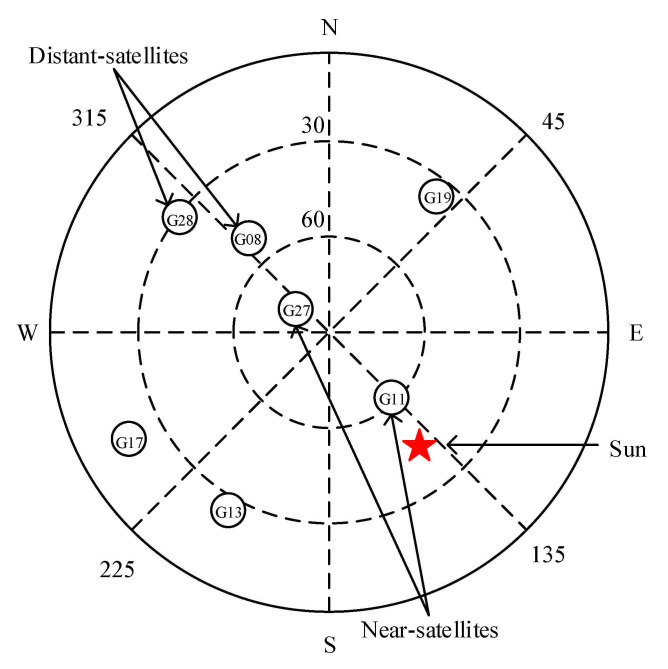
Sky image of IGS station KUNM at 03:30:00 (UTC) on 13 December 2006.

Figure 8 shows a sky image of the KUNM station at 03:30:00 (UTC) on 13 December 2006. The star-marked point represents the position of the Sun. The near-satellites G11 and G27 are consistent with the direction of the elevation of the Sun, while the distant-satellites G08 and G28 are in opposite directions.

Table 6 lists the azimuth (Az) and elevation (El) of the Sun and the satellites at 03:30:00 (UTC). Two near-satellites and two distant-satellites are selected for each station.

There are few satellites that meet the requirements of ‘‘near-satellites’’ or ‘‘distant-satellites’’, and thus, the values of $N_{sat}$ = 3 and $N_{station}$ = 2 from the optimization in Section 4.1 and Section 4.2 cannot be used for this section. This part of the experiment includes the following two steps:

- For a single station, the intersection of the valley periods of two near-satellites (distant-satellites) is taken.
- For each SRB event, the valley period common to at least two stations is judged as the final detection result.

**Figure 9:**
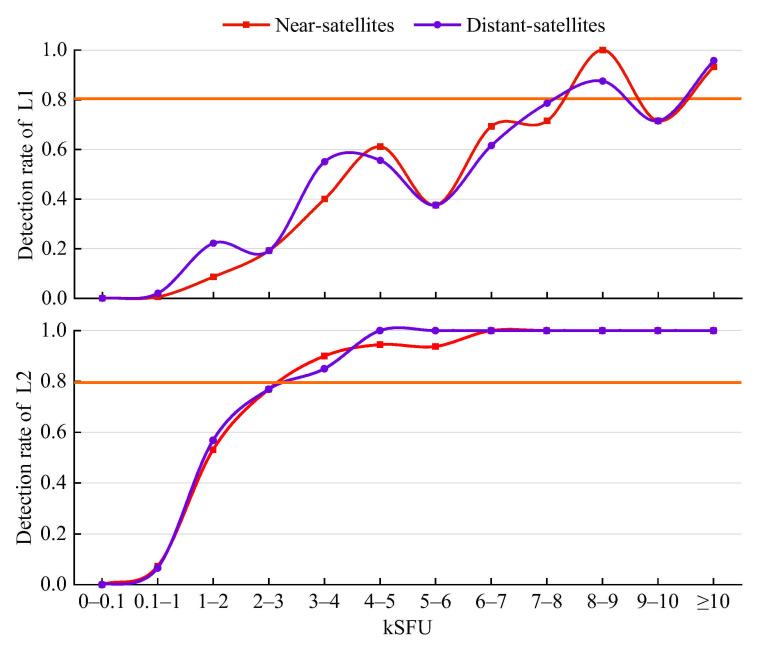
Influence of satellite distribution on the detection rate for 4 typical intense L-band SRB events in Table 5.

The detection results of each flux range for four typical intense L-band SRB events in Table 5 are shown in Figure 9. For GPS L1, the detection results of the near-satellites and distant-satellites are different in several flux ranges. The results for the two categories are generally equivalent. Specifically, the detection rate of the distant-satellites is higher in the ranges of 1–2 kSFU, 3–4 kSFU, and 7–8 kSFU but lower in the ranges of 4–5 kSFU, 6–7 kSFU, and 8–9 kSFU. For GPS L2, the differences between the detection rate of the near-satellites and that of the distant-satellites are −3.70%, 5.00%, −5.56%, and −6.25%, respectively, which are smaller than those at the L1 frequency. Moreover, the detection rate at the L2 frequency is much smoother than that at the L1 frequency. Consequently, the distribution of the satellites relative to the Sun has no effect on the overall detection rate.

**Figure 10:**
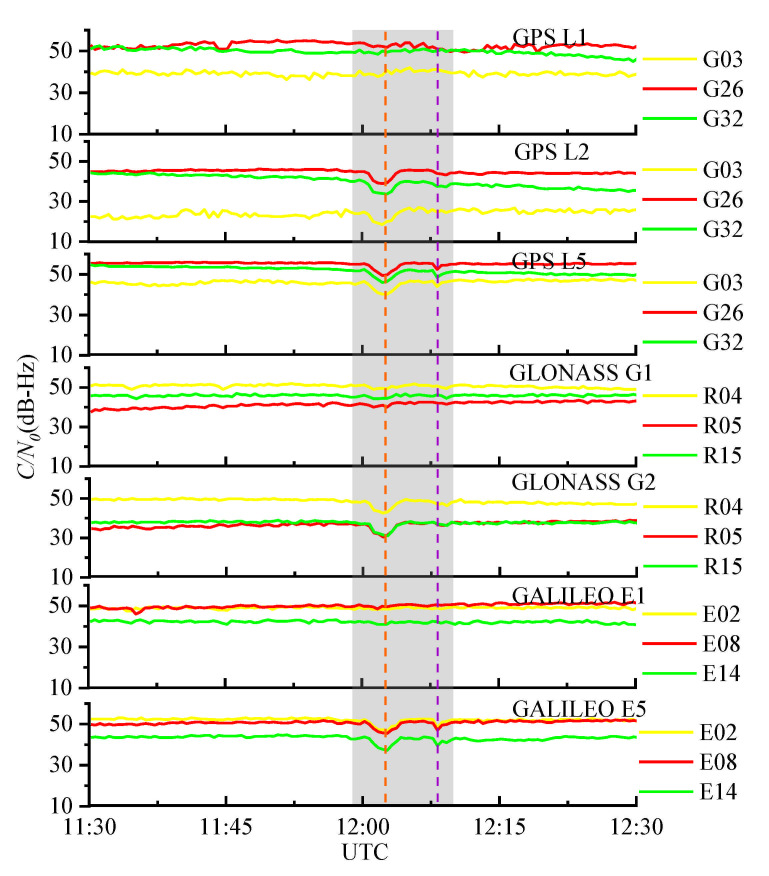
The $C/N_{0}$ values of different GNSS systems at IGS station HARB during an intense L-band SRB event that occurred on 6 September 2017. The sampling period was 30 s. The elevation mask angle was 10°.

Figure 10 presents the carrier-to-noise ratio observation data of GPS, GLONASS, and Galileo at the HARB station during 11:30:00–12:30:00 (UTC) on 6 September 2017, and the shaded area indicates the period when the SRB occurred. At the time of the peak flux density (12:02:30), the $C/N_{0}$ values of GPS L2, GPS L5, GLONASS G2 and Galileo E5 drop sharply by approximately 5–7 dB-Hz. Additionally, there is a slight drop near 12:08:00 (UTC). In each subdiagram of Figure 10, the decline degree of $C/N_{0}$ at each satellite appears to be similar at the same frequency. However, the $C/N_{0}$ values at various frequencies are affected by the SRB to different extents. The $C/N_{0}$ values of the high frequencies of GNSS (GPS L1, GLONASS G1, and Galileo E1) show an inconspicuous decrease, which is consistent with the conclusions of the study in [22]. They also pointed out that a possible reason for this is the different sensibilities of the GNSS frequencies and systems to SRB interference. Accordingly, this phenomenon leads to a difference in the detection rates at different GNSS frequencies in subsequent experiments.

**Figure 11:**
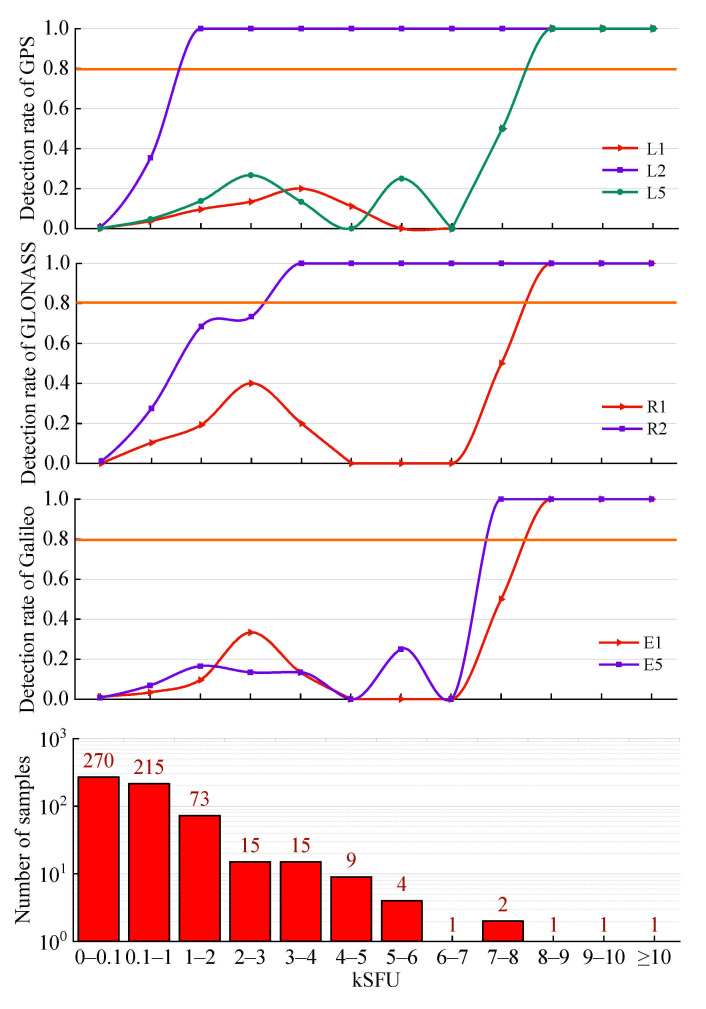
Detection results of different GNSS systems at various frequencies during an intense L-band SRB event that occurred on 6 September 2017.

Figure 11 shows the detection results at different frequencies for different systems. The detection performance at GPS L2 is the best of all systems. Specifically, the detection rates at GPS L2 of the flux ranges ≥ 1 kSFU are 100%, with a low false alarm rate. Moreover, the detection performance at GLONASS G2 is second only to that at GPS L2 and achieves 100% on the condition of flux ranges ≥ 3 kSFU.

Overall, a comparison of Figure 10 and Figure 11 shows that the more obvious the response to the SRB system is, the better the detection performance is. The detection thresholds of GPS L1 L5, GLONASS G1, and GALILEO E1 E5, under the condition of a detection rate above 80%, are 8–9 kSFU, 8–9 kSFU, 8–9 kSFU, 8–9 kSFU, and 7–8 kSFU, respectively, indicating worse performance than other frequencies and systems. However, the responses of the $C/N_{0}$ values of GPS L5 and GALILEO E5 to SRB is still evident, which appears to contradict the above trend. Through ample analysis, we found that the data of GPS L5 and GALILEO E5 from the IGS contain few satellites that can be observed simultaneously (the values vary from 4 to 6 and 5 to 8, respectively). Hence, the values of $N_{sat}$ = 3 and $N_{station}$ = 2 from the optimization in Section 4.1 and Section 4.2 are not suitable for GPS L5 and GALILEO E5. Through specific experiments, the optimal values for $N_{sat}$ and $N_{station}$ for GPS L5 are 2 and 2, respectively. For GALILEO E5, the values are 1 and 4, respectively. Although we modified the optimal values for $N_{sat}$ and $N_{station}$ for GPS L5 and GALILEO E5 to adapt to their smaller number of satellites and obtain better performance, the detection performance still appears to be unsatisfactory, as shown in Figure 11. The reason is that the core step of the proposed method is the comparison of multiple satellites and multiple stations, and thus, a reduction in the number of satellites can have a great impact on the detection results.

Sato et al. [21] determined the impacts of the SRB at the GPS L2 and L5 frequencies but not at the L1 frequency during the SRB event on 6 September 2017. Beyond their work, our analysis indicates that this SRB event may have an impact at the L1 frequency. The impact of this SRB event on the $C/N_{0}$ at the L1 frequency is difficult to observe with the naked eye but can be reflected by the experimental results of the proposed method. As shown in Figure 11, the detection rate is much higher in the ranges above 100 SFU than at 0–100 SFU. Although the number of samples for 6–7 kSFU, 8–9 kSFU, 9–10 kSFU, and ≥10 kSFU is merely 1, the number of samples in other ranges is sufficient. The detection rate of 0–100 SFU is close to 0%, while the number of samples in this range accounts for almost half of all samples. Most of the predicted samples are in flux density ranges above 100 SFU, and this phenomenon does not seem to be an accident.

## 5. Conclusions

Based on the influence of intense L-band SRBs on the $C/N_{0}$ of GNSS, a new method for detecting intense L-band SRB events is proposed. The influence of the number of satellites, number of monitoring stations, satellite distribution and different GNSS systems on the detection results are analyzed through several experiments. The conclusions can be summarized as follows:

1. The detection rate of intense L-band SRBs reaches more than 80% for the flux density above 800 SFU at the L2 frequency of GPS.
2. The detection results of GPS L2 and GLONASS G2 are better than those of GPS L1 L5, GLONASS G1 and Galileo E1 E5.
3. The distribution of the satellites relative to the Sun has no impact on the overall detection rate.
4. Statistically, the proposed detection algorithm is proven to have high reliability, with a false alarm rate of approximately 0% for historical SRB events detection with the optimal $N_{sat}$ and $N_{station}$.

The integrity, continuity, availability and accuracy of GNSS are important evaluation indexes for navigation satellite systems, and the interference of solar radio noise is a critical factor affecting the performance of GNSS. With the rapid development of GNSS, it is necessary to assess the negative impact of intense L-band SRBs on the system. Dispensing with expensive radio telescopes, we propose an intense L-band SRB detection method with high recognition rate, low false alarm rate, low cost, and all-weather real-time monitoring. The proposed method and insights in this paper can be conducive to the identification of intense L-band SRBs, which is a key component of attempting to suppress SRBs interference on GNSS in the future.

## Figures and Tables

**Table 1:** Directive characteristics of a navigation receiver antenna.

Elevation Angle, *β*°	G(β)in (dB)	$D(\beta )=P(\beta )/P_{0}$
0<β<5	−7.5≤G(β)≤−5	0.1775≤D(β)≤0.316
5<β<15	−4.5≤G(β)	0.354≤D(β)
β>15	−2≤G(β)	0.63≤D(β)

**Table 2:** Antenna effective area.

Elevation Angle, *β*°	$A_{e}(\beta )\mathrm{in}\;(m^{2})$	
	λ = 0.190 in (m) (L1)	λ = 0.244 in (m) (L2)
0<β<5	$\begin{array}{c} 5.099\times 10^{-4}\leq A_{e}(\beta )\\ \leq 9.077\times 10^{-4} \end{array}$	$\begin{array}{c} 8.409\times 10^{-4}\leq A_{e}(\beta )\\ \leq 1.497\times 10^{-3} \end{array}$
5<β<15	$1.01\times 10^{-3}\leq A_{e}(\beta )$	$1.677\times 10^{-3}\leq A_{e}(\beta )$
β>15	$1.809\times 10^{-3}\leq A_{e}(\beta )$	$2.984\times 10^{-3}\leq A_{e}(\beta )$

**Table 3:** Rate of the solar radio emission flux.

$L_{n}\;=\;10\cdot \mathrm{lg}\left(P_{n}\right),\;\mathrm{dBW}$	Rate of the Solar Radio Emission Flux (k), SFU					
	1	10^2^	10^3^	10^4^	10^5^	10^6^
0<β<5	−187.1	−167.1	−157.1	−147.1	−137.1	−127.1

**Table 4:** The ID, latitude, longitude and country of the IGS stations used in the experiments.

ID	Latitude/°	Longitude/°	Country
ISPA	110 W	27 S	Chile
AREQ	72 W	16 S	Peru
BOGT	75 W	4 N	Colombia
MDO1	105 W	30 N	USA
CHPI	45 W	22 S	Brazil
CRO1	65 W	17 N	USA
KOUR	53 W	5 N	Guyana
NNOR	116 E	31 S	Australia
PERT	115 E	31 S	Australia
SUNM	153 E	27 S	Australia
TIDB	148 E	35 S	Australia
PIMO	121 E	14 N	Philippines
CCJM	142 E	27 N	Japan
KUNM	102 E	25 N	China
GUAM	144 E	13 N	Guam
USUD	138 E	36 N	Japan
TSKB	140 E	36 N	Japan
DARW	131 E	12 S	Australia
TOW2	147 E	19 S	Australia
RABT	7 W	33 N	Morocco
MAS1	16 W	27 N	Spain
SFER	7 W	36 N	Spain
VILL	4 W	40 N	Spain
YEBE	4 W	40 N	Spain
KOKB	160 W	22 N	USA
TLSE	1 E	43 N	France
MBAR	30 E	0 N	Uganda
HARB	27 E	25 S	Africa
EBRE	0 E	40 N	Spain
MATE	14 E	40 N	Italy
MAT1	14 E	40 N	Italy

**Table 5:** Information on four intense L-band SRBs. The solar radio emission power is obtained from different RSTN stations due to the incompleteness of the RSTN data.

Date	Period	RSTN Station	Peak Time	IGS Station ID and Solar Incident Angle/(°)						
06.12.06	12:11–20:24	Sagamore-Hill	19:30	ISPA: 83.5	AREQ: 58.3	BOGT: 50.6	MDO1: 37.6	CHPI: 34.8	CRO1: 34.5	KOUR: 32.3
13.12.06	00:00–10:00	Learmonth	03:30	NNOR: 77.1	PERT: 76.3	SUNM: 66.6	TIDB: 68.5	PIMO: 52.4	CCJM: 38.0	KUNM: 36.0
15.02.11	02:04–10:45	Learmonth	03:00	PIMO: 75.8	GUAM: 80.0	USUD: 72.6	TSKB: 72.2	DARW: 59.0	KUNM: 58.8	TOW2: 50.4
24.09.11	11:00–22:00	Sagamore-Hill	13:00	RABT: 56.2	MAS1: 63.0	CHPI: 53.4	SFER: 53.2	KOUR: 51.7	VILL: 48.8	YEBE: 48.8

**Table 6:** Location information of the Sun and selected satellites at seven stations at 03:30:00 (UTC) on 13 December 2006.

Station	Sun		Distant-Satellites			Near-Satellites		
	Az/°	El/°	SVID	Az/°	El/°	SVID	Az/°	El/°
NNOR	80.9	77.1	G13	272	45	G11	5	52
			G23	215	66	G20	140	65
PERT	78.9	76.3	G13	275	45	G11	7	52
			G23	217	66	G20	140	65
SUNM	279.8	66.6	G25	145	45	G20	210	60
			G31	140	35	G23	230	32
TIDB	301.2	68.5	G25	135	47	G20	220	70
			G31	135	40	G23	243	45
PIMO	169.9	52.4	G19	20	29	G11	210	80
			G27	305	70	G13	230	20
CCJM	196.3	38.0	G03	50	40	G11	230	50
			G19	10	55	G16	120	30
KUNM	151.8	36.0	G08	325	50	G11	135	60
			G28	310	28	G27	300	80

## Data Availability

Not applicable.
